# HDAC Inhibitors Correct Frataxin Deficiency in a Friedreich Ataxia Mouse Model

**DOI:** 10.1371/journal.pone.0001958

**Published:** 2008-04-09

**Authors:** Myriam Rai, Elisabetta Soragni, Kai Jenssen, Ryan Burnett, David Herman, Giovanni Coppola, Daniel H. Geschwind, Joel M. Gottesfeld, Massimo Pandolfo

**Affiliations:** 1 Laboratoire de Neurologie Expérimentale, Hôpital Erasme, Université Libre de Bruxelles (ULB), Brussels, Belgium; 2 Department of Molecular Biology, The Scripps Research Institute, La Jolla, California, United States of America; 3 Program in Neurogenetics, Department of Neurology, David Geffen School of Medicine, University of California Los Angeles, Los Angeles, California, United States of America; National Institutes of Health, United States of America

## Abstract

**Background:**

Friedreich ataxia, an autosomal recessive neurodegenerative and cardiac disease, is caused by abnormally low levels of frataxin, an essential mitochondrial protein. All Friedreich ataxia patients carry a GAA⋅TTC repeat expansion in the first intron of the frataxin gene, either in the homozygous state or in compound heterozygosity with other loss-of-function mutations. The GAA expansion inhibits frataxin expression through a heterochromatin-mediated repression mechanism. Histone modifications that are characteristic of silenced genes in heterochromatic regions occur at expanded alleles in cells from Friedreich ataxia patients, including increased trimethylation of histone H3 at lysine 9 and hypoacetylation of histones H3 and H4.

**Methodology/Principal Findings:**

By chromatin immunoprecipitation, we detected the same heterochromatin marks in homozygous mice carrying a (GAA)_230_ repeat in the first intron of the mouse frataxin gene (KIKI mice). These animals have decreased frataxin levels and, by microarray analysis, show significant gene expression changes in several tissues. We treated KIKI mice with a novel histone deacetylase inhibitor, compound **106**, which substantially increases frataxin mRNA levels in cells from Friedreich ataxia individuals. Treatment increased histone H3 and H4 acetylation in chromatin near the GAA repeat and restored wild-type frataxin levels in the nervous system and heart, as determined by quantitative RT-PCR and semiquantitative western blot analysis. No toxicity was observed. Furthermore, most of the differentially expressed genes in KIKI mice reverted towards wild-type levels.

**Conclusions/Significance:**

Lack of acute toxicity, normalization of frataxin levels and of the transcription profile changes resulting from frataxin deficiency provide strong support to a possible efficacy of this or related compounds in reverting the pathological process in Friedreich ataxia, a so far incurable neurodegenerative disease.

## Introduction

Friedreich ataxia (FRDA, OMIM 229300) is an inherited recessive disorder characterized by progressive neurological disability and heart disease [1]. Onset is usually in childhood, but it may vary from infancy to adulthood. Atrophy of sensory and cerebellar pathways causes ataxia, dysarthria, fixation instability, deep sensory loss and loss of tendon reflexes. Corticospinal degeneration leads to muscular weakness and extensor plantar responses. With progression, patients lose the ability to walk and become dependent for all activities. In some cases, visual loss and neurosensorial deafness further increase disability. A hypertrophic cardiomyopathy, present in most cases, may become symptomatic and even cause premature death. Other common problems include kyphoscoliosis, pes cavus, and, in 10 % of patients, diabetes mellitus [1].

FRDA is caused by partial deficiency of the mitochondrial protein frataxin. Though the function of frataxin is still partly controversial, there is general agreement that it is involved in cellular iron homeostasis and that its deficiency results in multiple enzyme deficits, mitochondrial dysfunction and oxidative damage [2], [3]. Frataxin binds ferrous iron through negatively charged amino acids on its surface [4], it promotes the mitochondrial synthesis of iron-containing molecules, in particular iron-sulfur clusters (ISCs) [5] and heme [6], and it controls the ability of iron to perform redox chemistry [7]. Frataxin deficiency significantly affects ISC synthesis and results in reduced activities of several enzymes that require ISCs as prosthetic groups [8]. Frataxin may also have a more general protective effect against oxidative stress and in determining antioxidant responses, even in the absence of excess iron.

Complete absence of frataxin is incompatible with life in higher organisms, as demonstrated by the embryonic lethality observed in systemic gene knock-out models [9]–[12] and by the eventual loss of cells targeted for frataxin gene deletion in conditional knock-out models [13]. The human disease is caused by the pathological hyperexpansion of a GAA⋅TTC repeat sequence, ranging from 60–1700 repeats, in the first intron of the frataxin (*FXN,* MIM: 606829, GeneID: 2395) gene that partially suppresses *FXN* gene expression [14]. This mutation is present at the homozygous state in most patients and in compound heterozygosity with a different loss-of-function mutation in a small minority of cases (about 5%). In FRDA patients, frataxin amounts vary between 5% and 30% of those of normal individuals, and are little more than 50% of normal in heterozygous FRDA carriers, who have no sign of disease [14]–[16]. These findings suggest that restoring *FXN* gene expression in FRDA patients to heterozygote levels may substantially slow the course of the disease. In order to develop treatments to reduce or eliminate *FXN* transcriptional silencing, it is necessary to understand the underlying mechanisms. *In vitro* and in bacterial plasmids, pathological lengths of GAA repeats adopt a non-B, triple helical DNA structure that blocks and sequesters the advancing RNA polymerase [17]–[20]. The same repeats, when linked to a reporter gene in transgenic mice [21] and in cells from FRDA patients [22], become associated with transcriptionally silent heterochromatin. Therefore, decondensing the chromatin structure at the GAA repeat expansion appears an appealing target for FRDA therapeutics. Since deacetylated histones are generally associated with silent heterochromatin, HDAC inhibitors (HDACI) have the potential to make heterochromatin revert to an open, active conformation that allows gene expression [23]. In a previous study, a substantial increase in frataxin in lymphoblastoid cells and in primary, non-replicating lymphocytes from FRDA patients was only obtained when using a specific class of HDACI, analogs of the compound BML-210 (*N^1^*-(2-aminophenyl)-N^8^-phenyloctanediamide), including the highly active compound **4b** (*N* ^1^-(2-aminophenyl)-*N* ^7^-phenylheptanediamide) [22]. **4b** and related compounds raised frataxin levels to and above those of carriers, under conditions without apparent toxicity. The increase in *FXN* transcription is accompanied by increased acetylation of histone H3 at lysine14 (H3K14), as well as at H4K5 and H4K12 near the GAA repeat [22].

As a further step to evaluate these drugs as potential FRDA therapeutics, we have now investigated the efficacy and acute toxicity of a compound from this novel family of HDACI in a FRDA mouse model. Our data indicate that in a mouse model that carries expanded GAA repeats in the endogenous frataxin (*fxn*) gene, a member of this class of HDACI is able to restore frataxin levels and the gene expression profile to those of wild-type mice.

## Results

### fxn ^(GAA)230/(GAA)230^ (KIKI) mice recapitulate the genetic and epigenetic features of FRDA and have an altered gene expression profile as a consequence of mild frataxin deficiency

**Figure 1:**
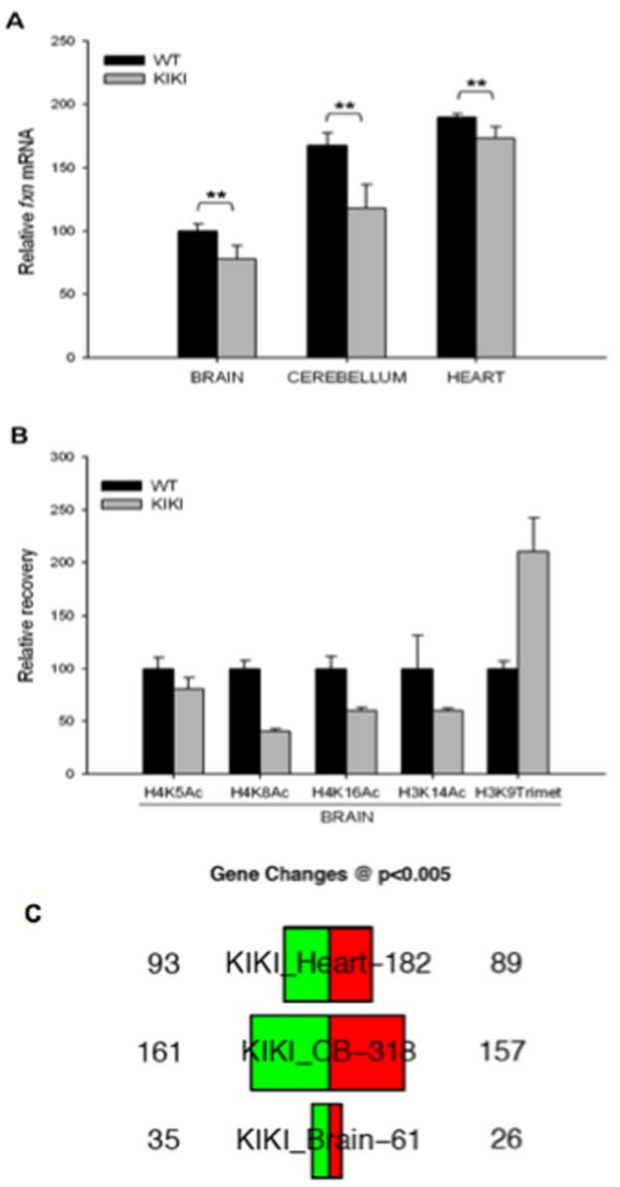
Frataxin mRNA levels and histone modifications on chromatin in the first intron of the frataxin gene in KIKI and WT mice. (A) We determined frataxin mRNA levels by quantitative real-time RT-PCR in brain, heart and cerebellum of 8 weeks old WT and KIKI mice (n = 8). All values are normalized to RER1 and β2m mRNA levels and are relative to WT brain, set to 100. Error bars are SD. Data were generated in two independent experiments. (** *P*<0.001). (B) Levels of H4K5, H4K8, H4K16, H3K14 acetylation and of H3K9 trimethylation in KIKI and WT mice brains (n = 8). We performed ChIP experiments with antibodies for murine histones H3 and H4 that carry each of the indicated modifications. We used primer pairs from the first intron of the mouse frataxin gene just upstream of the point of insertion of the GAA repeat in KIKI mice. Relative recovery, determined by quantitative real-time RT-PCR, is expressed in relation to GAPDH and the recovery in samples from WT animals is set to 100 for each antibody. Error bars are SEM. of four independent immunoprecipitations, each quantified in triplicate. (C) Histogram showing the number of genes differentially expressed in KIKI mouse brain (cerebral hemispheres), cerebellum and heart compared to WT mice. Genes with increased expression in KIKI mice are in red, genes with decresed expression in KIKI mice are in green. Only genes showing changes at P<0.005 are counted.

We previously generated a knock-in mouse carrying a (GAA)_230_ repeat in the first intron of the endogenous frataxin gene (*fxn*) [24], and we showed that homozyogous *fxn* ^(GAA)230/(GAA)230^ (KIKI) mice have mildly but significantly lower frataxin levels than wild-type (WT) animals with the same strain background (C57Bl/6), as determined by densitometry of western blots and semiquantitave RT-PCR [24]. In the present study, we confirmed by quantitative real-time RT-PCR the reduction in frataxin mRNA in KIKI brain, cerebellum and heart (Figure 1A). We then investigated the histone H3 and H4 post-translational modifications in chromatin near the GAA repeat by chromatin immunoprecipitation (ChIP). We found changes that correspond to those occurring in cells from FRDA patients [22], though they are quantitatively less marked, as expected given the relatively short GAA repeats in KIKI mice compared to a typical FRDA patient (Figure 1B). Taken together, these findings validate the KIKI mouse as a model for the frataxin transcriptional silencing and corresponding epigenetic changes as observed in FRDA.

Though KIKI frataxin levels are not expected to cause an overt phenotype, as they even exceed the 50% expression found in asymptomatic gene knockout heterozygous mice [10], we investigated whether they cause changes in gene expression profiles in tissues that are affected in FRDA, such as the central nervous system (CNS) and heart. For this purpose, we performed high-density microarray studies to compare the global gene expression profile in the brain (cerebral hemispheres), cerebellum and heart of KIKI mice to wild-type mice. 615 unique genes were found to be differentially expressed in KIKI vs WT samples at *P*<0.005 (Figure 1C). Fold changes were small in amplitude (within 2-fold changes). Complete gene lists are in Table S1 and have been deposited in the GEO database (accession number GSE10745). KIKI mice therefore have a clear, reproducible molecular phenotype. The biological relevance of this phenotype is further indicated by the significant overlap with gene expression changes found in FRDA patients' lymphocytes and in more severely frataxin-deficient *fxn* ^(GAA)230/-^ (KIKO) mice [25], [26]. In the absence of any detectable pathology, and therefore of non-specific changes due to cell atrophy and degeneration, these gene expression modifications are likely to represent a direct compensatory or stress-related response to lower levels of frataxin that can be used as a biomarker to evaluate the effectiveness of attempts to restore normal frataxin levels.

### Effects of compound 106 on frataxin levels and histone modifications in KIKI mice

**Figure 2:**
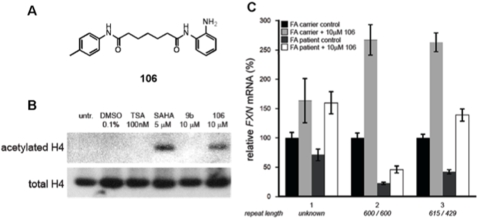
Effects of HDACI 106 on histone acetylation and *FXN* mRNA. (A) Structure of HDACI 106. (B) Histone H4 acetylation in normal fibroblast cells (GM08333 Coriell Cell Repositories, Camden, NJ) was measured by Western blot of total cell lysate after 24 hr incubation with indicated compounds. 9b (*N* ^1^-(2-methoxyphenyl)-*N* ^7^-phenylheptanediamide) has been described [22]. Compounds were dissolved in DMSO prior to addition to cell culture to give the final concentration indicated above at 0.1% DMSO. (C) *FXN* mRNA levels were measured in RNA from isolated PBMCs from carrier/patient pairs (1–3) after a 48 hour incubation with 106 or DMSO (at 0.1%). Quantitative real-time RT-PCR was used to determine relative *FXN* levels between each carrier/patient pair using GAPDH as a control housekeeping gene. Error is represented as the standard deviation of the mean from 3 determinations. Primary lymphocytes were obtained from donor blood from FRDA patients and their carrier relatives (under an approved Human Subjects Protocol, with appropriate informed consent) as described [22].

**Figure 3:**
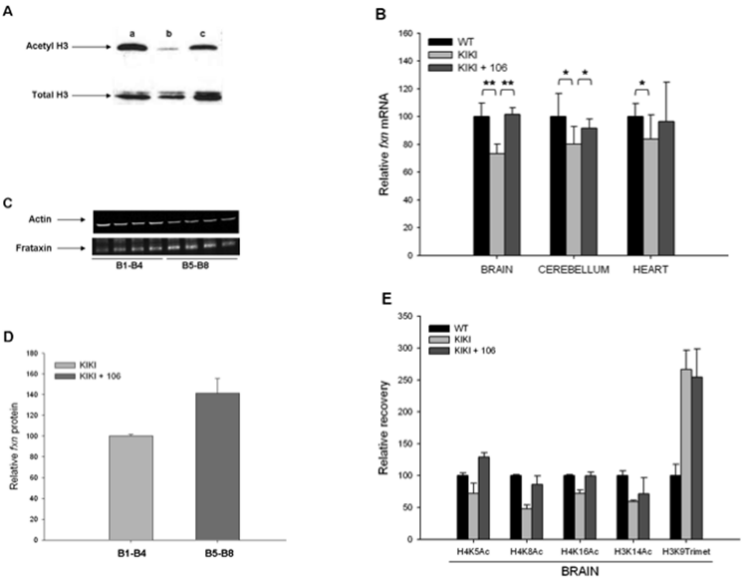
Effects of HDAC inhibitor 106 on frataxin mRNA and protein levels and on histone acetylation in KIKI mice. (A) The effect 106 on histone acetylation persists for at least 24 hours in KIKI mice. Mice were sacrificed, then extraction of brain acid-soluble nuclear proteins, SDS-PAGE, and western blotting with antibodies to total histone H3 and to acetylated histone H3 were performed. Sample in lane a is from the brain of a 106-treated mouse sacrificed after four hours, in lane b is from the brain of a vehicle-treated mouse, in lane c is from the brain of a 106-treated mouse sacrificed after 24 hours. (B) Frataxin mRNA levels in WT mice and in KIKI mice treated with either vehicle or HDAC inhibitor 106. Frataxin levels were determined by quantitative real-time RT-PCR relative to RER1 and β2m mRNAs, both unaffected by the HDACI. The WT frataxin mRNA level in each tissue was set to 100. Brain: n = 12. Cerebellum and Heart: n = 8. Data from 3 separate experiments were pooled and bars indicate SD. (** *P*<0.001; * *P*<0.05). (C) Western blot of frataxin in brain extracts of KIKI mice that had been treated with daily injections of 150 mg/Kg of 106 (lanes B5–B8) or vehicle (lanes B1–B4) for three days. Mice were sacrified 24 hours after the last injection. Actin was detected for normalization. (D) Densitometric analysis of the frataxin western blot shown in (C). (E) Levels of H4K5, H4K8, H4K16 and H3K14 acetylation of H3K9 trimethylation in KIKI mice treated with one subcutaneous injection per day of 150 mg/kg of 106 for 3 consecutive days compared to vehicle-treated KIKI littermates and WT (n = 6). We performed ChIP experiments with antibodies for murine histones H3 and H4 carrying each modification. Primer pairs corresponded to the first intron of the mouse frataxin gene just upstream of the point of insertion of the GAA repeat in KIKI mice. Relative recovery, determined by quantitative real-time PCR, is expressed in relation to GAPDH and the recovery in samples from WT animals is set to 100 for each antibody. Error bars are SEM of four independent immunoprecipitations, each quantified in triplicate.

Compound **106** (*N* ^1^-(2-aminophenyl)-*N* ^7^-*p*-tolylheptanediamide; see Figure 2A for structure and Methods S1 for synthetic methods), a derivative of **4b**, was found to be highly active in increasing histone acetylation in cell culture and *FXN* mRNA levels in primary lymphocytes from FRDA patients (Figure 2, B and C). Moreover, the parent compound **4b** and other derivatives increased histone acetylation in the brains of WT mice when injected subcutaneously (data not shown). Based on these results, we next examined whether treatment of KIKI mice with **106** resulted in increased histone acetylation in the brain, and we estimated the duration of this effect. After three doses of 150 mg/Kg of **106** given subcutaneously once a day for three consecutive days, increased histone acetylation was evident in the brain at least for 24 hours after the last injection, but had completely disappeared after a week (Figure 3A). Next, a group of 12 KIKI mice received subcutaneous injections of 150 mg/kg of **106** every 24 hours for three consecutive days to evaluate the effects of the compound on *fxn* mRNA and frataxin protein levels and on histone modifications near the GAA repeat. No apparent animal toxicity was associated with **106** treatment. Tissues (brain, cerebellum, and heart) were processed for RNA and protein extraction and for chromatin immunoprecipitation (ChIP) experiments.

*Fxn* mRNA, quantified by quantitative real-time RT-PCR, was significantly lower in the brain, cerebellum and heart of vehicle-treated KIKI mice than in similarly treated WT animals. Treatment with **106** increased KIKI *fxn* mRNA to levels that did not significantly differ from WT (Figure 3B), thus demonstrating correction of *fxn* deciciency in these animals. We confirmed that increased *fxn* mRNA levels resulted in higher frataxin protein level by western blotting (Figure 3, C and D). Treatment with **106** did not result in increased *fxn* mRNA levels in WT animals (Figure S1), indicating that its effect was due to removal of the inhibition caused by the GAA expansion.

We then checked whether in KIKI mice, as observed in lymphocytes from FRDA patients, **106** treatment was accompanied by changes in histone modifications in chromatin near the GAA repeat that could lead to a more transcription-permissive state. After HDACI treatment, trimethylation of H3K9 remained high, as previously observed in FRDA cells [24], but acetylation levels increased at all other explored positions in histones H3 and H4 (Figure 3E).

### Effect of HDACI 106 on the global gene expression profile in the brain, cerebellum and heart of WT and KIKI mice

**Figure 4:**
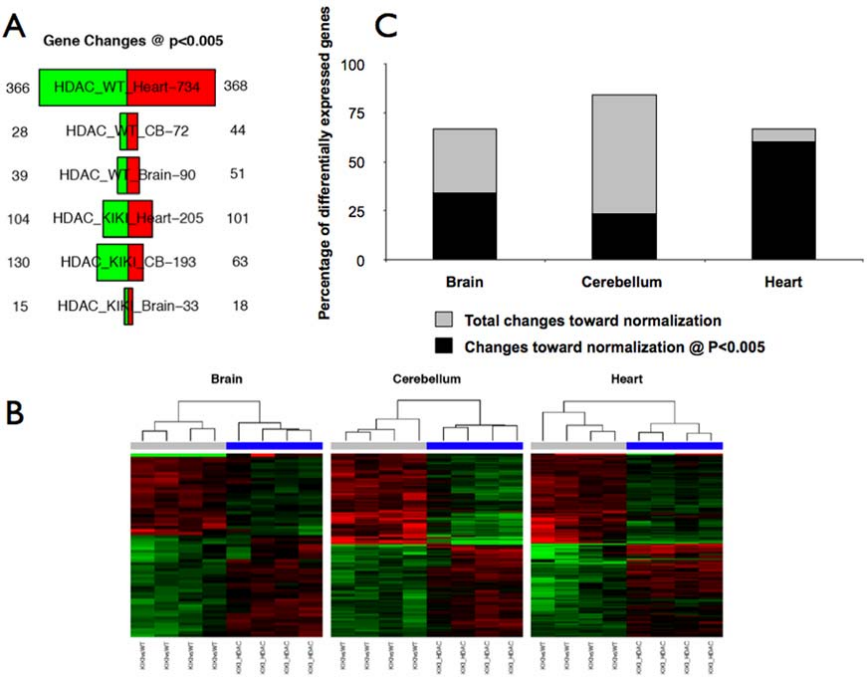
Effect of HDACI 106 on global gene expression profiles in the brain, cerebellum and heart of KIKI mice. (A) 670 probes (655 unique genes) in total were affected by drug treatment in either tissue in either strain. A few drug-responsive genes were shared between the two strains. Quantification of the number of genes (among the 615 KIKI-related) that go in opposite direction after drug treatment show that 70–85% genes change but only in KIKI samples. In a subset of genes (mostly in cerebellum) this difference was statistically significant at *P*<0.005. (B) Heatmaps representing the gene expression profiling before and after treatment with 106 in brain (left), cerebellum (center) and heart (right) of KIKI mice (four replicates per condition). Fold changes of the genes differentially expressed in KIKI vs. WT (brain = 61, cerebellum = 318, and heart = 182 genes at *P*<0.005) are depicted before treatment (KIKI vs. WT, grey bars on the top) and after treatment (KIKI treated vs. KIKI untreated, blue bars on the top). Shades of red represent upregulation, shades of green downregulation. Genes and samples are clustered by similarity. (C) Most genes (67% in brain, 84% in cerebellum, 67% in heart) show coordinate changes towards normal levels after treatment. In a subset of these genes (34% in brain, 23% in cerebellum, 60% in heart) these changes reach statistical significance.

Figure 4A shows the effects of treatment with **106** on gene expression in the brain, cerebellum and heart of KIKI and WT mice evaluated by high-density microarray analysis [27]. Complete gene lists are in Table S1 and are available from the GEO database (accession number GSE10745). 670 probes (655 unique genes) were affected by **106** treatment in either tissue in either strain, showing small (less than two-fold) changes in expression. The relatively limited overall effect of **106** on expression profiles and the lack of major changes in apoptosis or tumorigenesis-related genes suggest that severe adverse effect from this compound are unlikely, at least at the utilized dosage.

Despite some overlap, likely to reflect the general effects of this HDACI on gene expression, **106**-induced modifications of expression profiles significantly differed between WT and KIKI mice. The most striking finding concerned the set of genes that were differentially expressed in untreated KIKI vs. WT mice. Most of these genes (67% in brain, 84% in cerebellum, 67% in heart) showed coordinate changes towards normal levels after **106** treatment (Figure 4B). In a subset of these genes (34% in brain, 23% in cerebellum, 60% in heart at *P*<0.005; Table S1 and Figure 4C), these changes reached statistical significance. In WT mice, the same genes showed no significant change or minor changes in either direction. Therefore, treatment with **106** modified the global gene expression profile in KIKI animals to resemble the WT profile.

## Discussion

In the present study we have demonstrated the *in vivo* feasibility of a therapeutic approach to activate the *FXN* gene in a mouse model that recapitulates the genetic and epigenetic features of FRDA. Previous work has shown that *FXN* silencing in FRDA is likely to be the consequence of chromatin changes induced by the expanded intronic GAA repeat. Post-translational modifications of histone tails are thought to form a code, called the histone code [28], that affect gene expression by providing binding sites for proteins involved in controlling chromatin condensation and transcription. Increased trimethylation at H3K9 and decreased acetylation at H3K14, H4K5, H4K8, H4K12 and H4K16 constitute hallmarks of silent heterochromatin and are found immediately upstream and downstream of the GAA repat expansion in cells from FRDA patients [22]. KIKI mice have similar changes, indicating that they are a suitable model for *in vivo* testing of treatments to alter histone modifications that may restore frataxin levels in FRDA. We chose a novel HDACI, compound **106**, for testing in the animal model. **106** has been developed as an analog of the compound BML-210, the first HDACI shown to be effective in increasing acetylation levels at critical histone residues near the GAA repeat and in restoring frataxin levels in cultured cells from FRDA patients [22]. In contrast, other common potent HDACIs, such as as suberoylanilide hydroxamic acid (SAHA), suberoyl bis-hydroxamic acid (SBHA), trichostatin A (TSA), and valproic acid (VPA) do not increase *FXN* gene expression in cells from FRDA patients. The molecular basis for why these compounds are ineffective, as compared to the pimelic diphenylamides, exemplified by **106**, is currently under investigation.

We have established that **106** penetrates the blood-brain barrier and increases histone acetylation in the brain at a dose that causes no apparent toxicity in WT C57Bl/6 or in KIKI mice. This compound was able to restore normal frataxin levels in the central nervous system and heart of KIKI mice, tissues that are relevant targets as they are involved in FRDA pathology. As no effect on frataxin levels was observed in similarly treated WT mice, we conclude that **106** directly interferes with the transcriptional repression mechanism triggered by the GAA repeat, which is thought to involve the induction of transcriptionally silent heterochromatin. Accordingly, the typical histone marks of heterochromatic regions that are present near the GAA repeat in KIKI mice were partially removed by treatment with **106**. In particular, acetylation increased with treatment at several lysine residues in histones H3 and H4, but no decrease in H3K9 trimethylation occurred. We propose that increased acetylation of H3K14 and of K5, K8 and K16 on H4, results in a more open, transcription permissive chromatin state despite persisting H3K9 trimethylation, because it interferes with binding of repressive proteins that recognize the trimethylated H3K9 mark, such as heterochromatin protein 1 (HP1) [29].

Restoring frataxin expression represents an important step toward a treatment for FRDA if it is followed by functional recovery of affected cells. KIKI mice do not show overt pathology or abnormal behavior, but we identified changes in the overall gene expression profiles in relevant tissues that constitutes an observable, reproducible and biologically relevant phenotype as well as a biomarker to monitor the effectiveness of treatments. Remarkably, after **106** treatment gene expression profiles showed a clear trend toward normalization. This phenomenon cannot be considered a non-specific consequence of HDACI treatment, because the involved genes were not significantly modified in treated WT mice, whose frataxin levels also remained stable. Normalization of the transcription profile changes induced by lowered frataxin provides strong support to a possible efficacy of this or related compounds in reverting the pathological process in FRDA, at least as long as major cell loss has not occurred. Based on our results, potential therapeutics may be developed for FRDA, a so far incurable neurodegenerative disease.

## Materials and Methods

### Animal procedures

GAA knock-in mice were generated and genotyped as described [24]. Age and gender matched WT littermates were used as controls. Mice were treated by subcutaneous daily injections with 150 mg/kg of HDACI **106** or its equivalent of vehicle, for 3 consecutive days. Brain, heart and skeletal muscle were recovered 24 hours after last injection. All procedures respected regulations and guidelines of the Belgian state and European Union and were approved by the local ethical committee (CEBEA).

### Quantitative real-time RT-PCR

Total RNA from brain stem and/or cerebellum was extracted by RNeasy Lipid Tissue Mini Kit (Qiagen) as recommended by the manufacturer. RNA from heart was extracted by RNeasy Fibrous Tissue Mini Kit (Qiagen). All RNA samples were treated with RNase-Free DNase Set (Qiagen) and quantified afterwards by measuring the optical density (NanoDrop ND-1000 Spectrophotometer, NanoDrop Technologies). Quality and purity of some samples were analyzed by gel electrophoresis on RNA assay chips (Experion System and StdSens analysis kit, BioRad Laboratories). We performed one step quantitative real-time PCR PCR using MultiScribe Reverse Transcriptase with Power SYBR Green (both from Applied Biosystems) on the ABI 7500 Fast Real Time PCR System (Applied Biosystems). Primers used for *Fxn* were 5′- CCTGGCCGAGTTCTTTGAAG-3′ and 5′- GCCAGATTTGCTTGTTTGG-3′. RNA was standardized relative to *RER1* and ®**2m** (β2 microglobulin), mRNA using qBase 1.3.4 (Jan Hellemans & Jo Vandesompele). Data are normalized to the *Fxn* mRNA level in WT mice ( = 100%).

### Microarray

Three tissues were studied (brain, cerebellum, heart) in 4 biological replicates, two genotypes (KIKI and WT), and two treatments (**106** and vehicle), for a total of 48 microarrays. Total RNA (200 ng) was amplified, biotinylated and hybridized on Illumina Mouse v1.1 Expression arrays as per manufacturer's protocol. Raw data was log transformed and checked for outliers. Inter-array Pearson correlation and clustering based on variance were used as quality-control measures. Quantile normalization was used, and analysis of differential expression was performed using a linear model fitting [27], at a threshold of *P*<0.005 for statistical significance. Briefly, a linear model was fitted across the dataset, contrasts of interest were extracted, and differentially expressed genes for each contrast were selected using an empirical Bayes test statistic[27]. Differentially expressed genes were classified according to gene ontology, using Bioconductor packages and online tools (DAVID/EASE, http://david.niaid.nih.gov/david/ease.htm). Pathway analysis was carried out using Ingenuity Pathway Analysis (Ingenuity Systems, www.ingenuity.com).

### Western blot analysis

Tissues were homogenized in T-PER tissue protein extraction reagent (Pierce) for total proteins extraction. Histones were purified by acid extraction as described in the protocols from Upstate. Primary antibodies were diluted in Odyssey blocking buffer (Li-Cor) for frataxin (Chemicon) and actin (Sigma) or in PBS for total and acetylated histones antibodies (Upstate). Infrared dye conjugated secondary antibodies (anti-rabbit IRdye800Cw and anti-mouse IRdye680 from Li-Cor) were used to detect and quantify the signal of frataxin/actin using a Li-Cor Odyssey imaging system. Horseradish Peroxidase (HRP) - conjugated secondary antibodies (Santa Cruz Biotechnology) were used to detect the signal of total and acetylated histones by chemiluminescence.

### Chromatin immunoprecipitation

ChIP was performed as described previously [30]. We adapted the technique for use on fresh brain tissue and were able to test 3 different residues per one hemisphere. For each immunoprecipitation, lysate was incubated with one of the following antibodies (Anti-acetyl-Histone H3 (Lys14): Upstate Biotechnology 07-353; Anti-acetyl-Histone H4 (Lys5): Upstate 07-327; Anti-Histone H4 (acetyl K8): Abcam ab-1760; Anti-acetyl-Histone H4 (Lys16): Upstate 07-329; Anti-trimethyl-Histone H3 (Lys9)**:** Upstate 07-442; Normal Rabbit IgG**:** Upstate 12-370. Immunoprecipitated samples were quantified by real-time PCR following the standard curve method. Primers used for the region upstream intronic GAA repeats of *Fxn* were 5′- ACGACAAAGTCTCCCACAGG-3′ and 5′- GTCCAACAAGGCTTGATTCC-3′. GAPDH primers were: 5′-TGGGTGGAGTGTCCTTTATCC-3′ and 5′-TATGCCCGAGGACAATAAGG-3′.

### Statistical analyses

Data are presented as mean±s.d. or s.e.m., or as percentages. The significance of the difference between groups was evaluated with the Student's *t*-test. *P*<0.05 was considered significant. (* *P*<0.05 unless otherwise noted).

## Supporting Information

Figure S1 Effect of HDACI 106 on frataxin mRNA levels in WT C56Bl6 mice. Mice were treated with either vehicle (n = 7) or HDAC inhibitor 106 (n = 7). Frataxin mRNA levels were determined in brain (hemispheres), cerebellum and heart by quantitative real-time RT-PCR relative to RER1 and β 2m mRNAs, both unaffected by the HDACI. In each tissue, the frataxin mRNA level in vehicle-treated animals was set to 100. No changes were observed after 106 treatment.
(0.04 MB TIF)

Methods S1 Supplemental synthetic methods for compound 106.
(0.02 MB DOC)

Table S1 Microarray data
(15.57 MB XLS)

## References

[1] Pandolfo M, Wells RD, Ashizawa T (2006). Friedreich ataxia.. Genetic Instabilities and Neurological Diseases..

[2] Pandolfo M (2002). Frataxin deficiency and mitochondrial dysfunction.. Mitochondrion.

[3] Pandolfo M, Waxman S (2007). Friedreich ataxia and related RNA loss-of-function disorders.. Molecular Neurology..

[4] O'Neill HA, Gakh O, Isaya G (2005). Supramolecular assemblies of human frataxin are formed via subunit-subunit interactions mediated by a non-conserved amino-terminal region.. J Mol Biol.

[5] Chen OS, Hemenway S, Kaplan J (2002). Inhibition of Fe-S cluster biosynthesis decreases mitochondrial iron export: evidence that Yfh1p affects Fe-S cluster synthesis.. Proc Natl Acad Sci U S A.

[6] Yoon T, Cowan JA (2004). Frataxin-mediated iron delivery to ferrochelatase in the final step of heme biosynthesis.. J Biol Chem.

[7] O'Neill HA, Gakh O, Park S, Cui J, Mooney SM (2005). Assembly of human frataxin is a mechanism for detoxifying redox-active iron.. Biochemistry.

[8] Rotig A, de Lonlay P, Chretien D, Foury F, Koenig M (1997). Aconitase and mitochondrial iron-sulphur protein deficiency in Friedreich ataxia.. Nat Genet.

[9] Anderson PR, Kirby K, Hilliker AJ, Phillips JP (2005). RNAi-mediated suppression of the mitochondrial iron chaperone, frataxin, in Drosophila.. Hum Mol Genet.

[10] Cossée M, Puccio H, Gansmuller A, Koutnikova H, Dierich A (2000). Inactivation of the Friedreich ataxia mouse gene leads to early embryonic lethality without iron accumulation.. Hum Mol Genet.

[11] Vazzola V, Losa A, Soave C, Murgia I (2007). Knockout of frataxin gene causes embryo lethality in Arabidopsis.. FEBS Lett.

[12] Ventura N, Rea SL, Handerson ST, Condo I, Testi R, Johnson TE (2006). C. elegans as a model for Friedreich Ataxia.. Faseb J.

[13] Simon D, Seznec H, Gansmuller A, Carelle N, Weber P (2004). Friedreich ataxia mouse models with progressive cerebellar and sensory ataxia reveal autophagic neurodegeneration in dorsal root ganglia.. J Neurosci.

[14] Campuzano V, Montermini L, Moltò MD, Pianese L, Cossée M (1996). Friedreich's ataxia: autosomal recessive disease caused by an intronic GAA triplet repeat expansion.. Science.

[15] Campuzano V, Montermini L, Lutz Y, Cova L, Hindelang C (1997). Frataxin is reduced in Friedreich ataxia patients and is associated with mitochondrial membranes.. Hum Mol Genet.

[16] Montermini L, Richter A, Morgan K, Justice CM, Julien D (1997). Phenotypic variability in Friedreich ataxia: role of the associated GAA triplet repeat expansion.. Ann Neurol.

[17] Grabczyk E, Usdin K (2000). The GAA*TTC triplet repeat expanded in Friedreich's ataxia impedes transcription elongation by T7 RNA polymerase in a length and supercoil dependent manner.. Nucleic Acids Res.

[18] Ohshima K, Kang S, Larson JE, Wells RD (1996). Cloning, characterization, and properties of seven triplet repeat DNA sequences.. J Biol Chem.

[19] Ohshima K, Montermini L, Wells RD, Pandolfo M (1998). Inhibitory effects of expanded GAA.TTC triplet repeats from intron I of the Friedreich ataxia gene on transcription and replication in vivo.. J Biol Chem.

[20] Sakamoto N, Chastain PD, Parniewski P, Ohshima K, Pandolfo M (1999). Sticky DNA: self-association properties of long GAA.TTC repeats in R.R.Y triplex structures from Friedreich's ataxia.. Mol Cell.

[21] Saveliev A, Everett C, Sharpe T, Webster Z, Festenstein R (2003). DNA triplet repeats mediate heterochromatin-protein-1-sensitive variegated gene silencing.. Nature.

[22] Herman D, Jenssen K, Burnett R, Soragni E, Perlman SL, Gottesfeld JM (2006). Histone deacetylase inhibitors reverse gene silencing in Friedreich's ataxia.. Nat Chem Biol.

[23] Di Prospero NA, Fischbeck KH (2005). Therapeutics development for triplet repeat expansion diseases.. Nat Rev Genet.

[24] Miranda CJ, Santos MM, Ohshima K, Smith J, Li L (2002). Frataxin knockin mouse.. FEBS Lett.

[25] Coppola G, Burnett R, Engelhardt M, Suberlak MN, Perlman SL (2007). Transcriptome analysis in Friedreich's ataxia identifies potential biomarkers related to treatment response.. Annals of Neurology.

[26] Coppola G, Engelhardt M, Suberlak MN, Wexler EM, Santos MM (2007). Functional genomic analysis of Friedreich's ataxia pathogenesis in vivo and in vitro.. Annals of Neurology.

[27] Smyth GK, Gentleman VCR, Dudoit S, Irizarry R, Huber W (2005). Limma: Linear models for microarray data.. Bioinformatics and Computational Biology Solutions using R and Bioconductor.

[28] Jenuwein T, Allis CD (2001). Translating the histone code.. Science.

[29] Stewart MD, Li J, Wong J (2005). Relationship between histone H3 lysine 9 methylation, transcription repression, and heterochromatin protein 1 recruitment.. Mol Cell Biol.

[30] Luo RX, Postigo AA, Dean DC (1998). Rb interacts with histone deacetylase to repress transcription.. Cell.

