# Supplementary figures and images for: HDAC Inhibitors Correct Frataxin Deficiency in a Friedreich Ataxia Mouse Model
Source: PLoS One. 2008 Apr 9;3(4):e1958. doi: 10.1371/journal.pone.0001958 (PMC2373517; doi:10.1371/journal.pone.0001958)

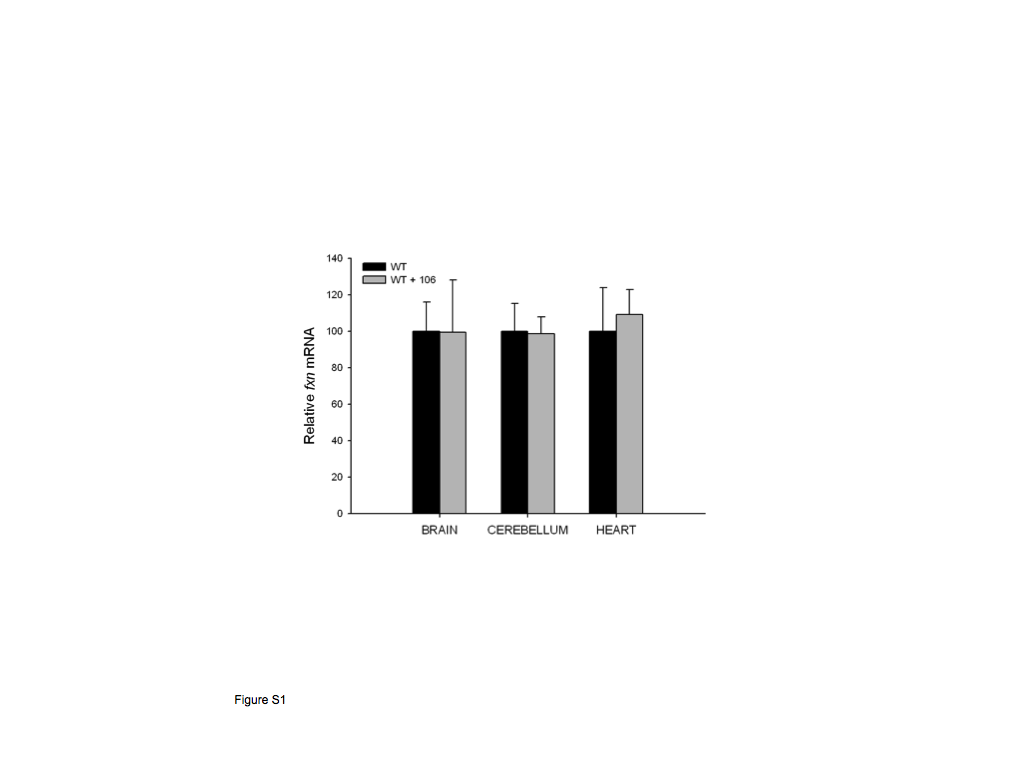

Supplement: Figure S1 — Effect of HDACI 106 on frataxin mRNA levels in WT C56Bl6 mice. Mice were treated with either vehicle (n = 7) or HDAC inhibitor 106 (n = 7). Frataxin mRNA levels were determined in brain (hemispheres), cerebellum and heart by quantitative real-time RT-PCR relative to RER1 and β 2m mRNAs, both unaffected by the HDACI. In each tissue, the frataxin mRNA level in vehicle-treated animals was set to 100. No changes were observed after 106 treatment. (0.04 MB TIF) [file pone.0001958.s001.tif]
