# Supplementary material for: HDAC Inhibitors Correct Frataxin Deficiency in a Friedreich Ataxia Mouse Model
Source: PLoS One. 2008 Apr 9;3(4):e1958. doi: 10.1371/journal.pone.0001958 (PMC2373517; doi:10.1371/journal.pone.0001958)
Supplement: Methods S1 — Supplemental synthetic methods for compound 106. (0.02 MB DOC) [file pone.0001958.s002.doc]

Supplemental synthetic methods

For synthesis of **106**, the precursor 7-oxo-7-(*p*-tolylamino)heptanoic acid was synthesized as follows: A solution of pimelic acid (2.50 g, 16.4 mmol) in acetic anhydride (10 mL) was heated under reflux for 20 minutes. After cooling to room temperature, the solvent was removed in vacuo. The crude yellow oil was used without any further purification for the next step. *p*-Toluidine (1.77 mL, 16.0 mmol) was added to a stirred solution of the produced anhydride in anhydrous THF (10 mL). After stirring at room temperature for 1 hour, the reaction mixture was diluted with water until a colorless solid precipitated, which was collected by filtration. Recrystallization from water/ethanol gave the pure compound as a colorless solid. This reaction yielded 1.56 g (6.3 mmol, 38 %): TLC: R*_f_* = 0.17 (DCM/MeOH 20:1); MS (MALDI-ToF, CHCA): *m/z* (%) = 250.3 (100, [M+H]^+^, calc. 250.1), 272.4 (24, [M+Na]^+^, calc. 272.1); ^1^H-NMR (500 MHz, D_4_-MeOH): δ 1.42-1.50 (m, 2H), 1.63-1.79 (m, 4H), 2.35 (s, 3H) 2.31 (d, ^3^*J*=7.5 Hz, 2H), 2.43 (d, ^3^*J*=7.6 Hz, 2H), 3.32 (s, 3H), 7.09-7.12 (AA’BB’, 2H), 7.48-7.51 (AA’BB’, 2H).

Synthesis of **106**, *N*^1^-(2-aminophenyl)-*N*^7^-*p*-tolylheptanediamide: A solution of the precursor described above (750 mg, 3.00 mmol), EDC•HCl (573 mg, 3.00 mmol), HOBt (460 mg, 3.00 mmol) in DMF (20 mL) was added dropwise to a stirred and cooled solution of 1,2-phenylenediamine (1.62 mg, 15.0 mmol) in DMF (10 mL). After stirring the reaction for 2 hours at room temperature, water was added until a white solid precipitated, which was collected by filtration and recrystallized from ethanol/water. This reaction yielded 742 mg (2.19 mmol, 73 %) of **106** as a colorless solid: TLC: R*_f_* = 0.53 (DCM/MeOH 20:1); MS (MALDI-ToF, CHCA): *m/z* (%) = 340.3 (100, [M+H]^+^, calc. 340.2), 362.4 (24, [M+Na]^+^, calc. 362.2); ^1^H-NMR (500 MHz, D_6_-DMSO): δ 1.33-1.36 (m, 2H), 1.60-1.65 (m, 4H), 2.25-2.33 (m, 4H), 3.35 (s, 3H), 4.80 (br. s, 2H), 6.50-6.54 (m, 1H), 6.70-6.72 (m, 1H), 6.87-6.90 (m, 1H), 7.07-7.09 (AA’BB’, 2H), 7.13-7.16 (m, 1H), 7.45-7.47 (AA’BB’, 2H), 9.05 (s, 1H), 9.76 (s, 1H). Analytical methods were as described^1^.

**Abbreviations**: CHCA (α-cyano-4-hydroxycinnamic acid), DCM (dichloromethane), diisopropylethylamine (DIPEA), dimethylsulfoxide (DMSO), dimethylformamide (DMF), *N*-ethyl-*N*’-(3-dimethylaminopropyl)carbodiimide hydrochloride (EDC•HCl), 1-hydroxy-1H-benzotriazole hydrate (HOBt), MeOH (methanol), room temperature (r.t), trifluoroacetic acid (TFA), and tetrahydrofurane (THF).

**Reference**

^1^Herman, D. et al. Histone deacetylase inhibitors reverse gene silencing in Friedreich's ataxia. *Nat Chem Biol* **2**, 551-8 (2006).
